# Cardiac-specific Trim44 knockout in rat attenuates isoproterenol-induced cardiac remodeling via inhibition of AKT/mTOR pathway

**DOI:** 10.1242/dmm.049444

**Published:** 2022-08-22

**Authors:** Xiao-yu Jiang, Fei-fei Guan, Jia-xin Ma, Wei Dong, Xiao-long Qi, Xu Zhang, Wei Chen, Shan Gao, Xiang Gao, Shuo Pan, Ji-zheng Wang, Yuan-wu Ma, Lian-feng Zhang, Dan Lu

**Affiliations:** 1 Key Laboratory of Human Disease Comparative Medicine, National Health Commission of China (NHC), Institute of Laboratory Animal Science, Chinese Academy of Medical Sciences, Peking Union Medical College, Beijing 100021, China; 2 Beijing Engineering Research Center for Experimental Animal Models of Human Critical Diseases, Institute of Laboratory Animal Science, Chinese Academy of Medical Sciences, Peking Union Medicine College, Beijing 100021, China; 3 National Human Diseases Animal Model Resource Center, Institute of Laboratory Animal Science, Chinese Academy of Medical Sciences, Peking Union Medicine College, Beijing 100021, China; 4 State Key Laboratory of Cardiovascular Disease, Fuwai Hospital, National Center for Cardiovascular Disease, Chinese Academy of Medical Sciences, Peking Union Medical College, Beijing 100037, China

**Keywords:** Trim44, Cardiac hypertrophy, Rat, Knockout, AKT/mTOR pathway

## Abstract

When pathological hypertrophy progresses to heart failure (HF), the prognosis is often very poor. Therefore, it is crucial to find new and effective intervention targets. Here, myocardium-specific Trim44 knockout rats were generated using CRISPR-Cas9 technology. Cardiac phenotypic observations revealed that Trim44 knockout affected cardiac morphology at baseline. Rats with Trim44 deficiency exhibited resistance to cardiac pathological changes in response to stimulation via isoproterenol (ISO) treatment, including improvement of cardiac remodeling and dysfunction by morphological and functional observations, reduced myocardial fibrosis and reduced expression of molecular markers of cardiac stress. Furthermore, signal transduction validation associated with growth and hypertrophy development *in vivo* and *in vitro* demonstrated that Trim44 deficiency inhibited the activation of signaling pathways involved in myocardial hypertrophy, especially response to pathological stress. In conclusion, the present study indicates that Trim44 knockout attenuates ISO-induced pathological cardiac remodeling through blocking the AKT/mTOR/GSK3β/P70S6K signaling pathway. This is the first study to demonstrate the function and importance of Trim44 in the heart at baseline and under pathological stress. Trim44 could be a novel therapeutic target for prevention of cardiac hypertrophy and HF.

## Abstract (precis)

**Summary:** This is the first study to demonstrate the function of Trim44 in the heart at baseline and under pathological stress. Trim44 could be a novel therapeutic target for prevention of cardiac hypertrophy.

## INTRODUCTION

Sustained pathological cardiac hypertrophy, which is characterized by increased weight and volume of the heart, cardiac contractility dysfunction and subsequent interstitial fibrosis, eventually progresses to heart failure (HF) (Diwan and Dorn, 2007; Lorell and Carabello, 2000; Zhang et al., 2015b). Extracellular stress, including biomechanical stress and neurohumoral mediators, activates intracellular signaling pathways, finally resulting in pathological cardiac hypertrophy. This process involves the expression of multiple genes with increased protein translation rate and decreased protein degradation rate. Therefore, it is important to find target molecules that selectively modulate signaling pathways involved in cardiac hypertrophy and HF (Heineke and Molkentin, 2006; van Berlo et al., 2013).

Tripartite motif-containing protein 44 (Trim44), which was first cloned in 2001 by Effrossini Boutou et al. (2001), has been identified as a member of the TRIM protein family. TRIM family proteins, most of which have E3 ubiquitin ligase activities, are involved in a variety of cellular processes, such as apoptosis, development, growth and signal transduction (Di Fiore et al., 2003; Hershko and Ciechanover, 1998; Yang et al., 2020; Chen et al., 2016, 2017; Prestes et al., 2016; Heliste et al., 2020; Zungu et al., 2011; Salazar-Mendiguchía et al., 2020). TRIM family proteins are also involved in multiple diseases, including those affecting innate immunity and neurodevelopment, cardiovascular diseases and carcinogenesis (Yang et al., 2013; Leong et al., 2002; Hatakeyama, 2011; Järvinen et al., 2008; Urano et al., 2009; Boutou et al., 2001).

Functional studies have demonstrated that different TRIMs, such as TRIM8, TRIM10, TRIM21, TRIM24, TRIM28, TRIM32, TRIM37, TRIM45, TRIM50, TRIM67, TRIM72 and TRIM76, have distinct effects on cardiac function and cardiac pathology, and are involved in cardiomyocyte apoptosis, ischemia–reperfusion injury, myocardial dysplasia and cardiomyopathy (Yang et al., 2020; Chen et al., 2017, 2012; Heliste et al., 2020; Borlepawar et al., 2019; Gupta et al., 2018; Brigant et al., 2018; Zhang et al., 2020; Rabinovich-Nikitin and Kirshenbaum, 2019; Zhang et al., 2021). Given the complexity of cardiac physiology and pathology, few TRIMs have been shown to have effects on the cardiovascular system. Ankush Borlepawar et al. evaluated the expression of all known TRIMs in myocardium in multiple cardiac diseases, such as cardiomyopathy, HF, atrial fibrillation and myocardial infarction (MI) (Borlepawar et al., 2019). Interestingly, in addition to known cardiac TRIMs, several other TRIMs were found to be differentially regulated in these disease conditions, and the expression of TRIM44 was increased under all the pathological conditions mentioned above. Accumulating evidence has shown that dysregulation of TRIM44 protein is associated with several diseases, such as cancer, developmental disorders, neurodegenerative disease, viral infection and aniridia (Yang et al., 2013; Yamada et al., 2020; Xie et al., 2021; Wei et al., 2019; Xiao et al., 2020; Peng et al., 2018; Li et al., 2018; Liu et al., 2018; Yu et al., 2021; Kashevarova et al., 2014; Zhang et al., 2015a; Samant et al., 2016; Lim et al., 2017; Landsend et al., 2018). Borlepawar et al.’s analysis of upregulated expression of TRIM44 in cardiac pathology suggests that it may be involved in the regulation of cardiovascular pathological processes. In addition, recent studies by Luo et al. have shown that TRIM44 is involved in cardiomyocyte injury in cultured human cardiomyocyte primary cells (Luo et al., 2020). However, how TRIM44 shapes heart morphology and function *in vivo* within the pathogenesis of hypertrophy or HF, especially in adults, remains unclear.

Therefore, to study the effect of Trim44 deficiency on heart development and its response to pathological stimulus and possible mechanisms, the Trim44 conditional knockout (cKO) rat was established using CRISPR-Cas9 technology. Trim44 was selectively eliminated in cardiomyocytes by crossing *Trim44* cKO rats with the *α-MHC* (also known as *Myh6*) rat, which was established in our previous study (Ma et al., 2017). Geometric and functional analyses of the heart revealed that Trim44 deficiency inhibited normal hypertrophic development. In addition, rats with Trim44 deficiency exhibited resistance to cardiac pathological changes in response to pathological hypertrophy stimulation via isoproterenol (ISO) treatment, with protection at the global, organizational and molecular levels. Furthermore, signal transduction validation *in vivo* and *in vitro* demonstrated that Trim44 deficiency in the myocardium inhibited the activation of cascade pathways involved in cardiac hypertrophy, AKT/mTOR/GSK3β/P70S6K, especially response to pathological stress. This is the first study to demonstrate the function and importance of Trim44 in the heart at baseline and under pathological stress.

## RESULTS

### Trim44 is expressed in myocardial cells and is induced under pathological cardiac hypertrophy and HF

**Fig. 1.**
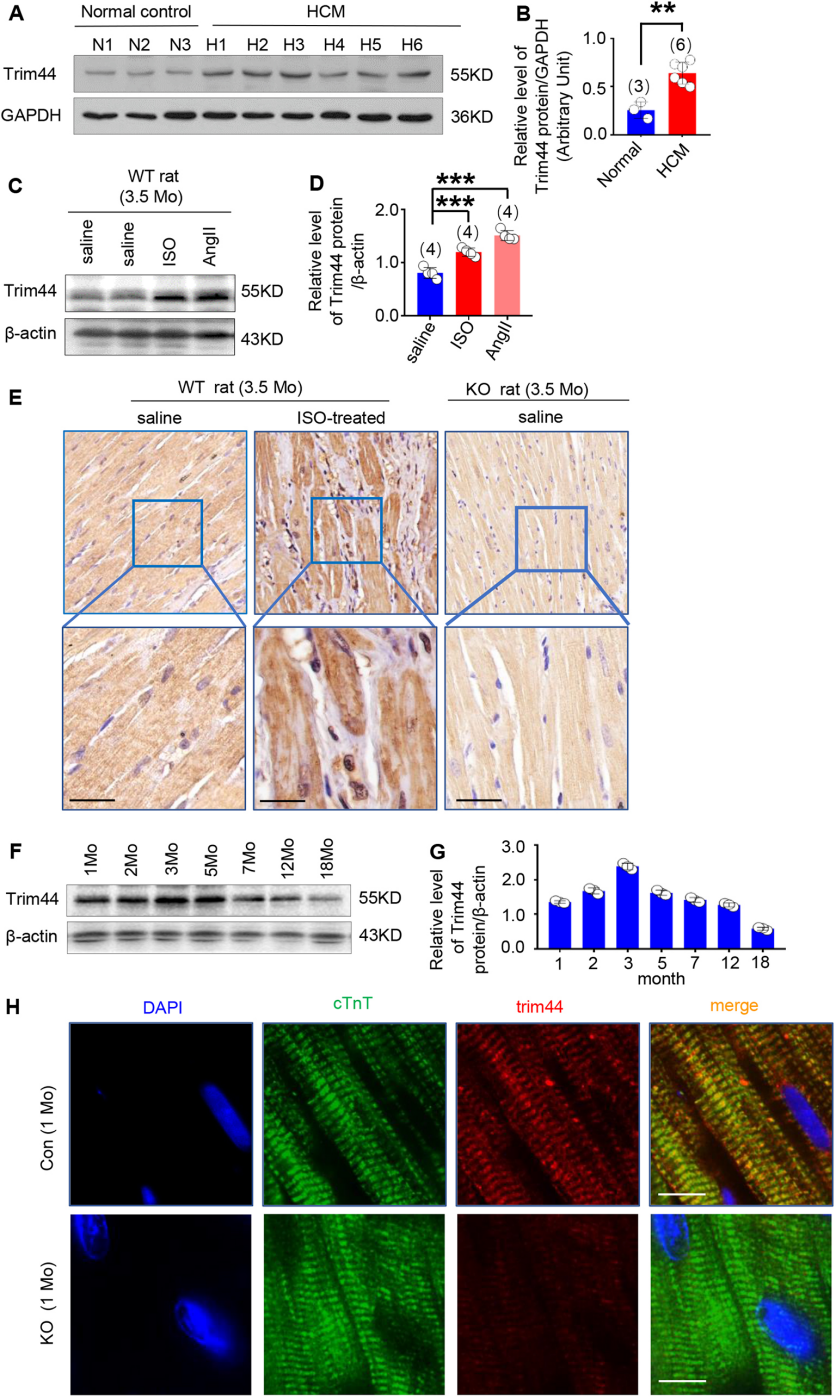
**Expression of Trim44 in myocardium under physiological and pathological conditions.** (A,B) Expression of TRIM44 in heart tissues from patients with hypertrophic cardiomyopathy (HCM) (*n*=6) and normal controls (*n*=3), detected using western blotting (A) and quantitatively analyzed using GAPDH for normalization (B). (C,D) Expression of Trim44 in heart tissues from rat models of cardiac hypertrophy/heart failure induced by isoprenaline (ISO) or angiotensin (ANG II) treatment, detected using western blotting (C) and quantitatively analyzed using β-actin for normalization (D) (*n*=4 rats per group). (E) Immunohistochemical analysis of Trim44 expression in paraffin sections of myocardial tissue from wild-type (WT) rats treated with ISO or saline and *Trim44* KO rats at 3.5 months of age (scale bars: 20 μm). (F,G) Expression of Trim44 in myocardium from control rats at 1, 2, 3, 5, 7, 12 and 18 months of age, detected using western blotting (F) and quantitatively analyzed using β-actin for normalization (G) (*n*=3 rats per time point). (H) Trim44 and cardiac troponin T (cTnT) immunofluorescence in paraffin sections of heart tissues from control and KO rats at 1 month of age (scale bars: 10 μm). **P<0.01, ***P<0.001 versus normal or saline group (unpaired two-tailed Student's *t*-test).

Borlepawar et al.’s analysis exhibited that the expression of TRIM44 increases under multiple pathological conditions (Borlepawar et al., 2019). Therefore, we evaluated the expression of TRIM44 in samples of patients with pathological cardiac hypertrophy. Analysis of the expression characteristics of TRIM44 in samples from hypertrophic cardiomyopathy (HCM) patients and healthy heart donors revealed that the expression of TRIM44 in heart tissues was increased significantly under HCM (Fig. 1A,B; *n*=3 in normal group, *n*=6 in HCM patient group, *P*<0.01, HCM group versus normal group). Furthermore, the expression of Trim44 was also increased obviously in ISO-induced or angiotensin (ANG II)-induced cardiac hypertrophy/HF rat models (Fig. 1C,D; *n*=4 rats per group, *P*<0.001, ISO- or ANG II-treated group versus the saline group). We further analyzed the expression of Trim44 in rat myocardial tissues of cardiac hypertrophy induced by ISO through immunohistochemical observation, and we also verified the lack of Trim44 staining in myocardial tissues from *Trim44* KO rats. Moreover, we found that Trim44 expression increased under pathological stimulation of cardiac hypertrophy and was mainly distributed in the cytoplasm (Fig. 1E).

Then, we evaluated the expression of Trim44 in heart tissues from wild-type (WT) rats. We monitored the expression of Trim44 over time, observing that Trim44 expression reached its peak in hearts of WT rats at 3 months of age, and decreased thereafter with age (Fig. 1F,G). Immunofluorescence analysis of paraffin sections of heart tissues revealed that Trim44 localized in cardiomyocytes but not in non-cardiomyocytes. In addition, we also performed immunofluorescence analysis of paraffin sections of *Trim44* KO heart tissues to verify the cardiomyocyte-specific loss of Trim44 (Fig. 1H).

The abnormally increased expression of Trim44 under pathological cardiac hypertrophy/HF states prompted us to further investigate the biological function of Trim44 in heart, especially in response to stress.

### Establishment of the myocardium-specific *Trim44* KO rat

**Fig. 2.**
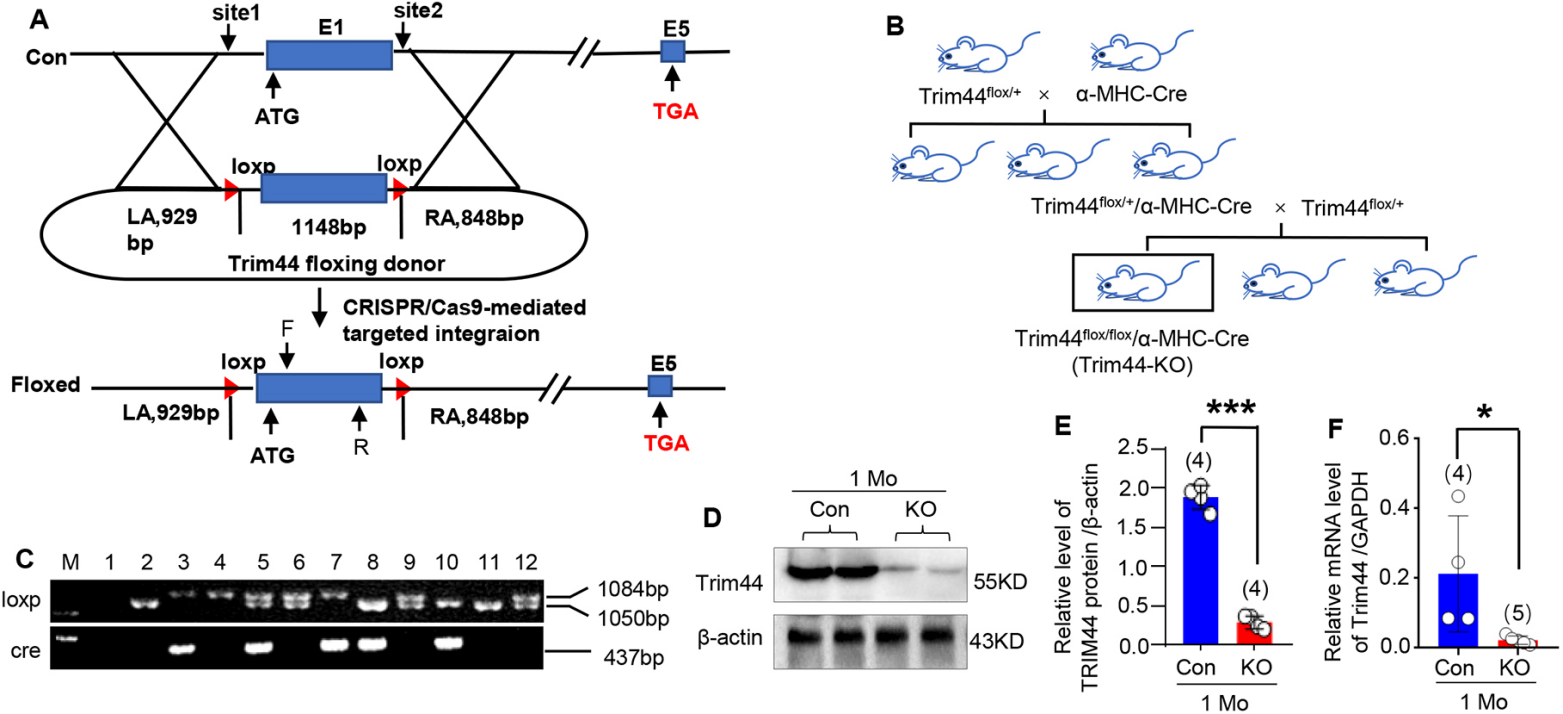
**Establishment of myocardial tissue-specific *Trim44* knockout rat.** (A) *Trim44* conditional knockout (cKO) rats were generated using CRISPR-Cas9 technology. (B) Myocardial tissue-specific *Trim44* knockout (KO) rats were generated by cross mating between the *Trim44* cKO rat and the *α-MHC-Cre* rat. (C) Gel electrophoresis of the genotyping (loxP band was 1084 bp, WT band was 1050 bp, and Cre band was 437 bp). Line M is molecular size ladder for electrophoresis, and lines 1-3 are negative control, standard sample from WT and KO rat, respectively. Sample in line 7 was determined as myocardial tissue-specific *Trim44* KO rat. (D,E) KO efficiency analysis of Trim44 protein in heart tissues from *Trim44* KO rats at 1 month of age, detected using western blotting (D) and quantitatively analyzed using β-actin for normalization (E) (*n*=4 rats per group). (F) Knockout efficiency analysis of Trim44 protein in heart tissues from *Trim44* KO rats at 1 month of age, detected using real-time PCR, using *Gapdh* for normalization (*n*=4-5 rats per group). *P<0.05, ***P<0.001 versus control group (unpaired two-tailed Student's *t*-test).

To explore how Trim44 shapes heart morphology and function *in vivo*, we first established *Trim44* cKO rats in this study (Fig. 2A). Trim44 was knocked out in cardiomyocytes by crossing a *Trim44* cKO rat with an *α-MHC* tool rat, which was established in our previous study (Ma et al., 2017) (Fig. 2B). Genotyping was carried out by PCR (Fig. 2C), and, after two rounds of cross breeding, the Mendelian ratio of *Trim44*^flox/flox^/*α-MHC-Cre* (referred to as *Trim44* KO) rat was ∼12.5%.

Furthermore, the expression level of Trim44 protein was confirmed by western blotting (Fig. 2D,E; *n*=4 rats per group, *P*<0.001, KO group versus control group). The knockout (KO) efficiency of the Trim44 protein reached up to 84.5% in total protein extract of whole-heart tissue from *Trim44* KO rats. In addition, the KO efficiency of *Trim44* reached 88.98% at mRNA level through RT-PCR (Fig. 2F; *n*=4 or 5 rats per group, *P*<0.05, KO group versus control group).

In addition, we determined the KO specificity of Trim44 in several organs from *Trim44* KO rats through western blotting. The results showed that Trim44 protein decreased specifically in heart tissues, not in other tissues, in *Trim44* KO rats (Fig. S1).

### Trim44 KO induces morphological abnormalities in adult rat heart

**Fig. 3.**
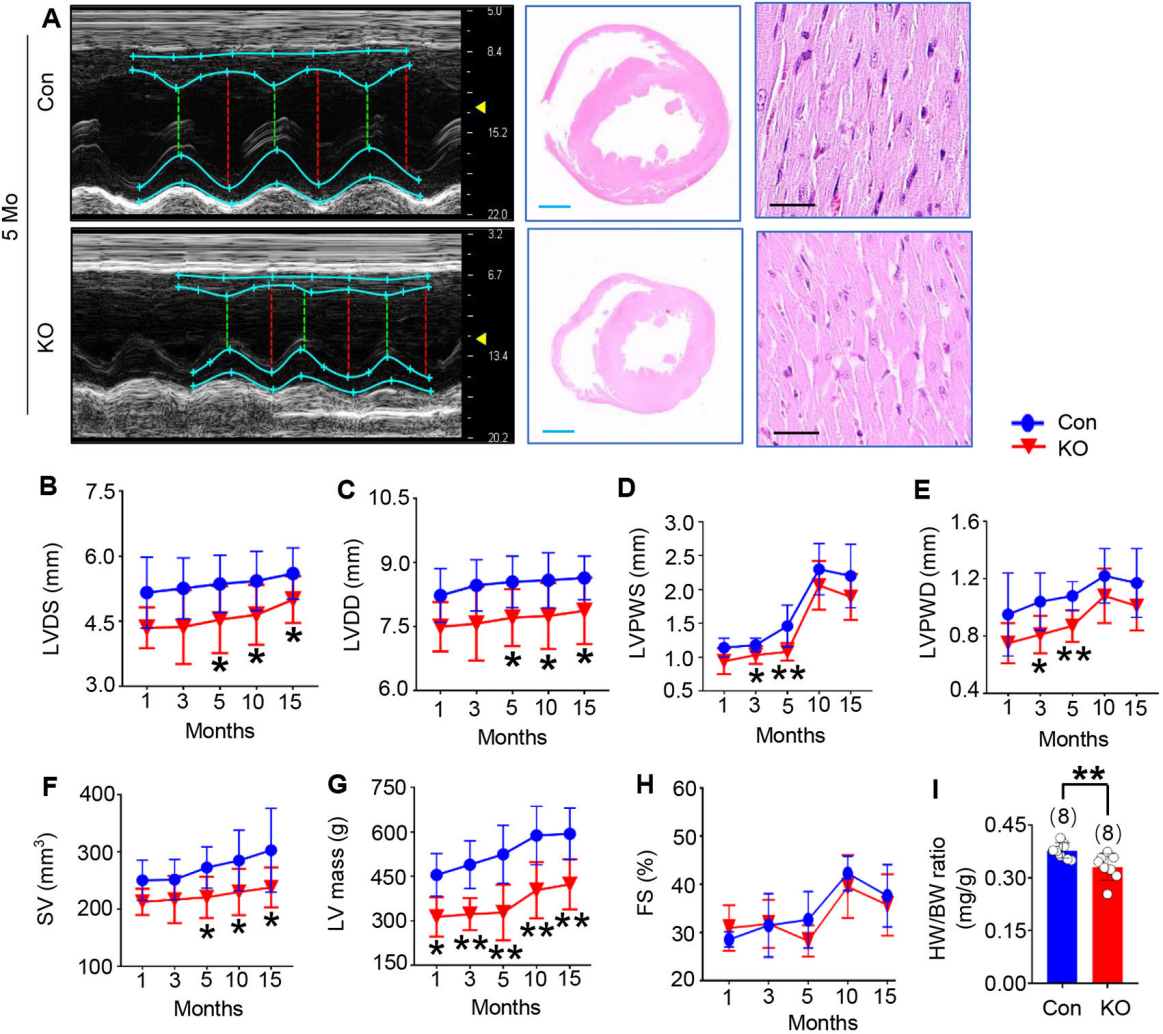
**Trim44 KO induces morphological abnormalities in adult rat heart.** (A) M-mode echocardiography screenshots (left column) and representative photographs of the whole-heart transverse section (middle column) and magnifications of the left ventricle (right column) with Hematoxylin and Eosin (H&E) staining of control and KO rats at 5 months of age (blue scale bars: 2 mm; black scale bars: 20 μm). (B-H) Echocardiographic parameters of left ventricular (LV) diameter at end systole (LVDS; B) and at end diastole (LVDD; C), LV posterior wall thickness at end systole (LVPWS; D) and at end diastole (LVPWD; E), stroke volume (SV; F), LV mass (G) and fractional shortening (H) were analyzed in KO rats at 1, 3, 5, 10 and 15 months of age (*n*=6-11 rats per time point in each group). (I) Heart weight to body weight ratio (HW/BW) in control and KO groups (*n*=8 rats per group). **P<0.01 versus control group (unpaired two-tailed Student's *t*-test).

We analyzed cardiac function and geometry using M-mode echocardiography in *Trim44* KO and control groups at 1, 3, 5, 10 and 15 months of age. The overall heart of the *Trim44* KO rats became smaller, with morphological changes occurring from 3 months of age, and exhibited typical characteristics at 5 months of age, including thinning of the ventricular wall, reduction of the ventricular cavity, decreased left ventricular (LV) mass and decreased stroke volume (SV) (Fig. 3A-G; Table S1). However, fractional shortening (FS) exhibited no difference between *Trim44* KO and control rats (Fig. 3H; *n*=6-11 rats per time point in each group). Furthermore, the ratio of heart weight to body weight (HW/BW) decreased significantly at 5 months of age (Fig. 3I; *n*=8 rats per group, *P*<0.01, KO group versus control group).

However, in both pups and adults, there were no differences in appearance or status between the control rats and *Trim44* KO rats, including body weight at 2 weeks, 5 months and 7 months of age (Tables S2-S4). In addition, we determined the cardiac phenotypes of *Trim44* KO rats at 2 weeks of age, and no difference was found in the echocardiographic analysis (Table S3). The diastolic function of the 7-month-old KO rats was determined, and the results showed no abnormal diastolic function (Table S4).

We next examined the phenotypes of *Trim44* KO rats at 7 months of age. The heart weight to tibial length ratio was reduced significantly. We detected the coefficients of several main organs, and the results showed that there were no obvious changes in organ coefficients except for the heart (Tables S4 and S5). In addition, Hematoxylin and Eosin (H&E) staining analysis of the main organs showed no obvious pathological changes (Fig. S2). These results suggested that only the structure and function of the heart were affected in KO rats, and no significant changes were observed in other main organs.

We also assessed the phenotypes of *Trim44* KO heterozygous rats at 7 months of age. Echocardiographic analysis indicated no significant changes in cardiac morphology and function. Moreover, heart weight to tibia length ratios and main organ coefficients of the heterozygous rats showed no differences compared with those of the control rats. The data are included in Tables S4 and S5.

### Trim44 KO attenuates morphology breakage and cardiac dysfunction in response to ISO-induced cardiac remodeling

In view of our observations above, Trim44 KO induced morphological and functional abnormalities in adult rat heart. However, the effects of Trim44 KO under pathological stress of cardiac hypertrophy/HF were still unknown. Therefore, we then subjected both the *Trim44* KO rats and control rats to ISO treatment to mimic pathological cardiac hypertrophy/HF stimulation (Kumari et al., 2020; Lu et al., 2013).

**Fig. 4.**
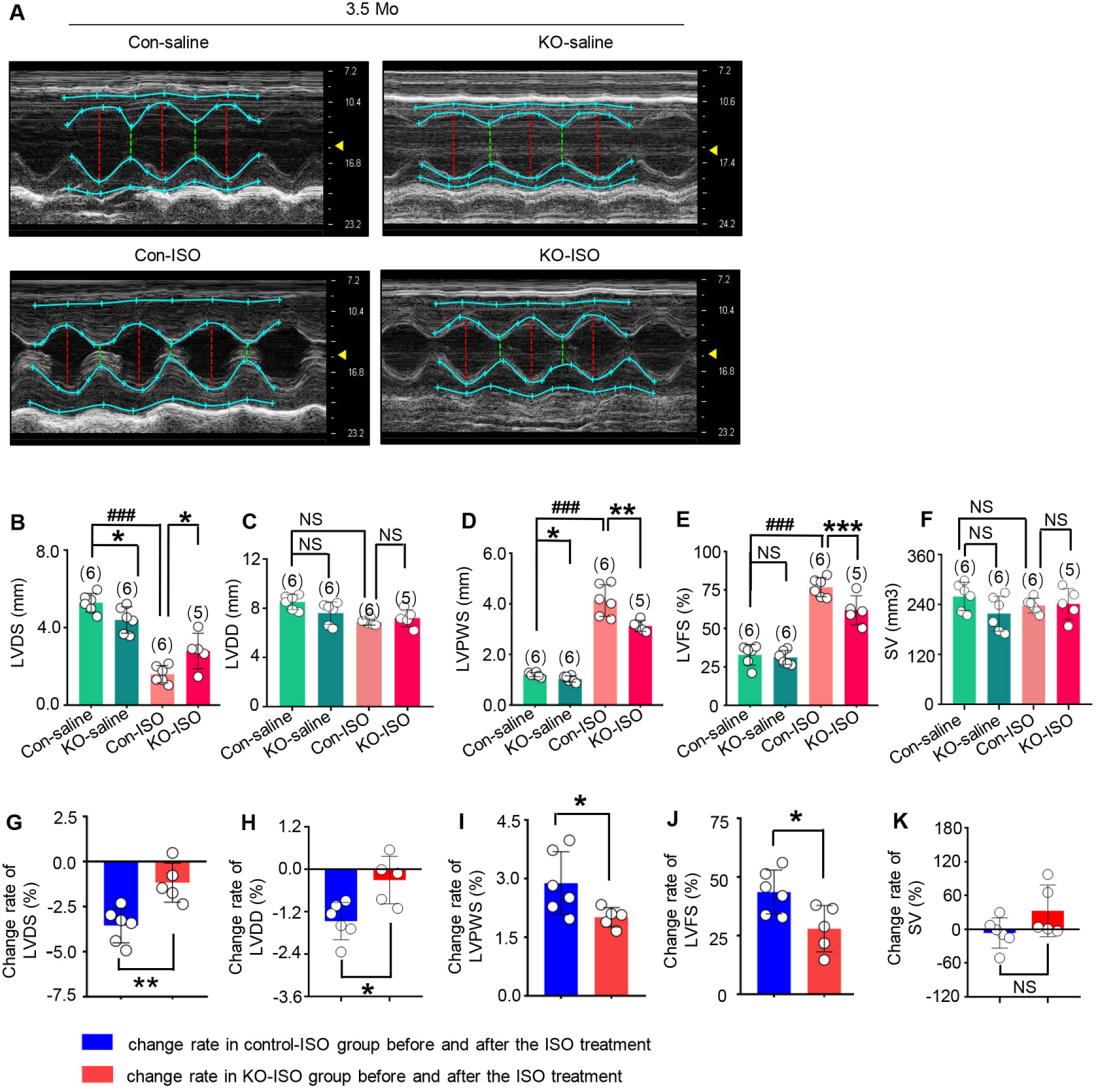
**Trim44 KO attenuates cardiac geometry destruction and dysfunction in response to ISO-induced cardiac remodeling.** (A) M-mode echocardiography screenshots of control-saline, KO-saline, control-ISO and KO-ISO rats at 3.5 months of age. (B-F) Echocardiographic parameters of LVDS (B), LVDD (C), LVPWS (D), LVFS (E) and LVSV (F) from the four groups at 3.5 months of age (*n*=5-6 rats per group). (G-K) The change rate of LVDS (G), LVDD (H), LVPWS (I), LVFS (J) and SV (K) in control-ISO and KO-ISO groups before and after ISO treatment. *P<0.05, **P<0.01, ***P<0.001, ^###^*P*<0.001 versus respective control group (unpaired two-tailed Student's *t*-test or one-way ANOVA). NS, not significant.

Morphological and functional changes of thick wall, small chamber and enhanced functionality were observed in ISO-treated control rats, as expected (Fig. 4A-E). These were demonstrated by the decreased LV diameter at end systole (LVDS) (Fig. 4A,B; *n*=6 rats per group, *P*<0.001, control-ISO versus control-saline), increased LV posterior wall thickness at end systole (LVPWS) (Fig. 4A,D; *n*=6 rats per group, *P*<0.001, control-ISO versus control-saline) and LVFS (Fig. 4A,E; *n*=6 rats per group, *P*<0.001, control-ISO versus control-saline) at the end of observation (2 weeks after cessation of ISO treatment). However, LV at end diastole (LVDD) and SV showed no differences between the control-ISO group and control-saline group (Fig. 4C,F).

Interestingly, *Trim44* KO rats exhibited a marked reduction in LV wall thickness and contractile function, and increased chamber diameter at the end of observation (2 weeks after cessation of ISO treatment) (Fig. 4A-E). Morphological parameters from echocardiographic analysis showed changes in the *Trim44* KO group without pathological stimulation; therefore, we analyzed echocardiographic parameters in the KO-ISO group and control-ISO group before and after the ISO treatment. We observed significant decreases in changes in LVDS, LVDD, LVPWS and LVFS (Fig. 4G-J; *P*<0.05, before and after the ISO treatment in the KO-ISO group and control-ISO group). However, the SV showed no difference in the KO-ISO and control-ISO groups before and after the ISO treatment (Fig. 4K).

After assessing changes in cardiac geometry and function, pathological changes in response to ISO-induced stress were further detected in the control-saline, KO-saline, control-ISO and KO-ISO groups of rats. Histopathological changes in gross morphology and myocardial microstructure were assessed by H&E staining, and the degree of fibrosis was detected by Masson's trichrome staining.

**Fig. 5.**
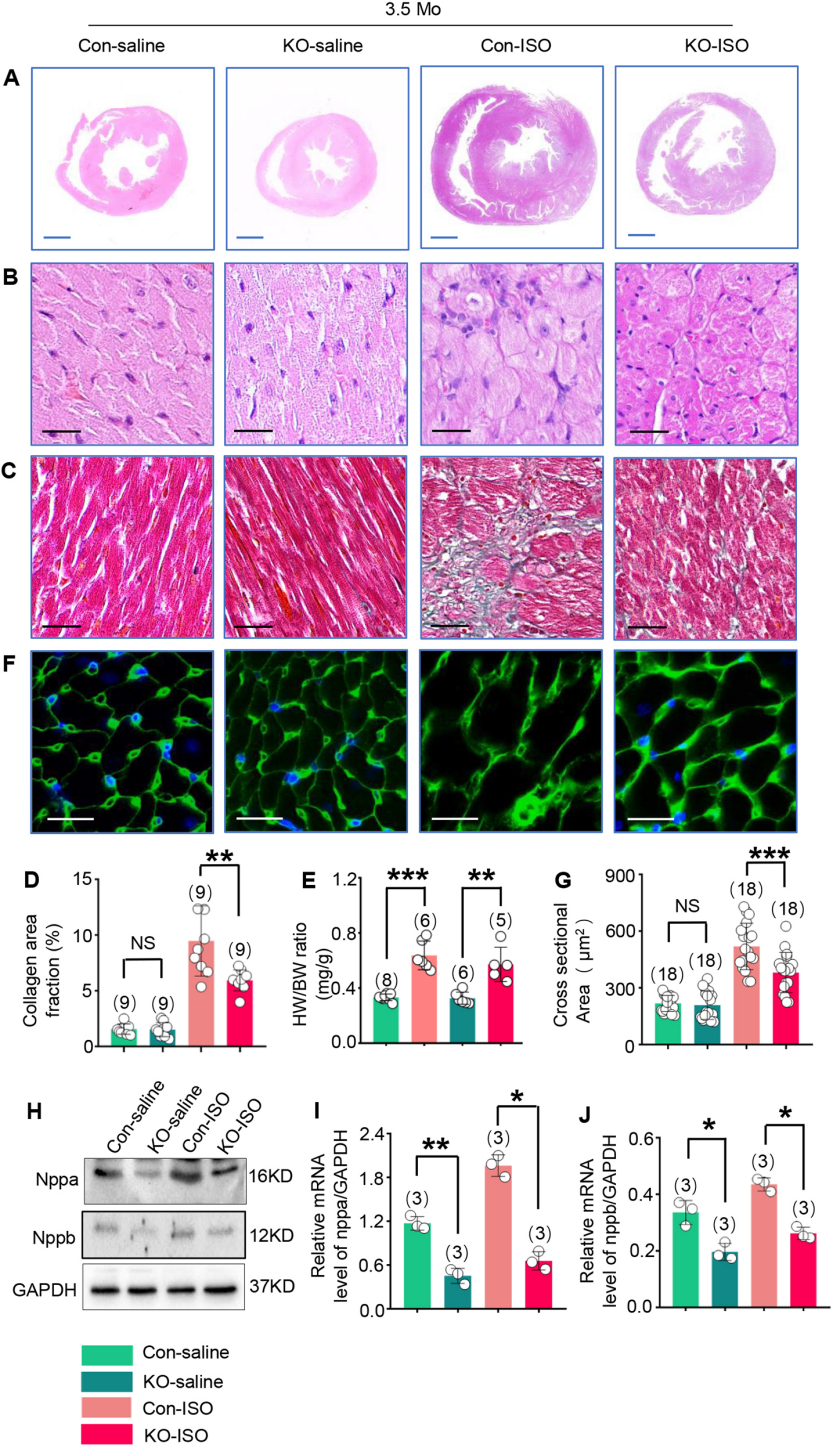
**Trim44 KO attenuates pathological changes in response to ISO-induced cardiac remodeling.** (A) Representative photographs of the whole-heart transverse sections with H&E staining from control-saline, KO-saline, control-ISO and KO-ISO groups 2 weeks after cessation of ISO treatment (blue scale bars: 2 mm). (B) Magnifications of left ventricle sections with H&E staining (black scale bars: 20 μm). (C) Magnifications of left ventricle sections with Masson trichrome staining (black scale bars: 20 μm). (D) Quantitative analysis of collagen area fraction in four groups (*n*=3 rats per group, three fields of views per rat). (E) HW/BW ratio in four groups (*n*=5-8 rats per group). (F) Magnifications of left ventricle sections with wheat germ agglutinin (WGA) immunofluorescence (white scale bars: 20 μm). (G) Quantitative analysis of cross-sectional area of cardiomyocytes in four groups (*n*=5-6 rats per group, 18 fields of view per group). (H-J) Expression of Nppa and Nppb, detected using western blotting (H), and quantitative analysis of *Nppa* (I) and *Nppb* (J) mRNA expression through real-time PCR, using *Gapdh* for normalization (*n*=3 rats per group). *P<0.05, **P<0.01, ***P<0.001 versus respective control group (one-way ANOVA). NS, not significant.

The thick wall, smaller chamber and malalignment in control-ISO rats were obviously attenuated by Trim44 KO in the KO-ISO group (Fig. 5A,B), which were all consistent with the results from gross morphology analysis by echocardiography. Collagen accumulation in the interstitial space, which was detected by Masson's trichrome staining, increased obviously in heart tissues from control-ISO rats, and decreased obviously in heart tissues from KO-ISO rats, compared with the control-ISO rats (Fig. 5C,D; *n*=3 rats per group, three fields of view per rat, *P*<0.01, KO-ISO versus control-ISO).

Furthermore, the HW/BW ratio increased by 91.5% in the control-ISO group compared with the control-saline group (Fig. 5E; *P*<0.001, control-ISO group versus control-saline group), while it only increased by 74.9% in the KO-ISO group compared with the KO-saline group (Fig. 5E; *P*<0.01, KO-ISO group versus KO-saline group), although this change still exhibited significance. Moreover, the cross-sectional area of cardiomyocytes detected by wheat germ agglutinin (WGA) immunofluorescence increased obviously in the heart tissues of control-ISO rats. This increase was attenuated significantly in the heart tissues of KO-ISO rats compared with the control-ISO rats (Fig. 5F,G; *n*=5-6 rats per group, 18 fields of view per group, *P*<0.001, KO-ISO group versus control-ISO group).

We further detected markers of cardiac stress, including natriuretic peptide A and B (Nppa and Nppb), through western blotting, and found significant downregulation of *Nppa* and *Nppb* mRNA expression in the heart tissues of KO-ISO rats compared with those of control-ISO rats (Fig. 5H-J; *n*=3-5 rats per group, *P*<0.05, KO-ISO versus KO-saline).

These results indicated that the deletion of Trim44 significantly inhibited the development of pathological hypertrophy and HF, including by improving overall heart morphology and function, reflected by echocardiographic parameters, as well as reducing myocardial fibrosis, hypertrophic cardiac cell size and levels of markers of cardiac stress.

### Trim44 KO attenuates ISO-induced cardiac remodeling through inhibition of the AKT/mTOR pathway

**Fig. 6.**
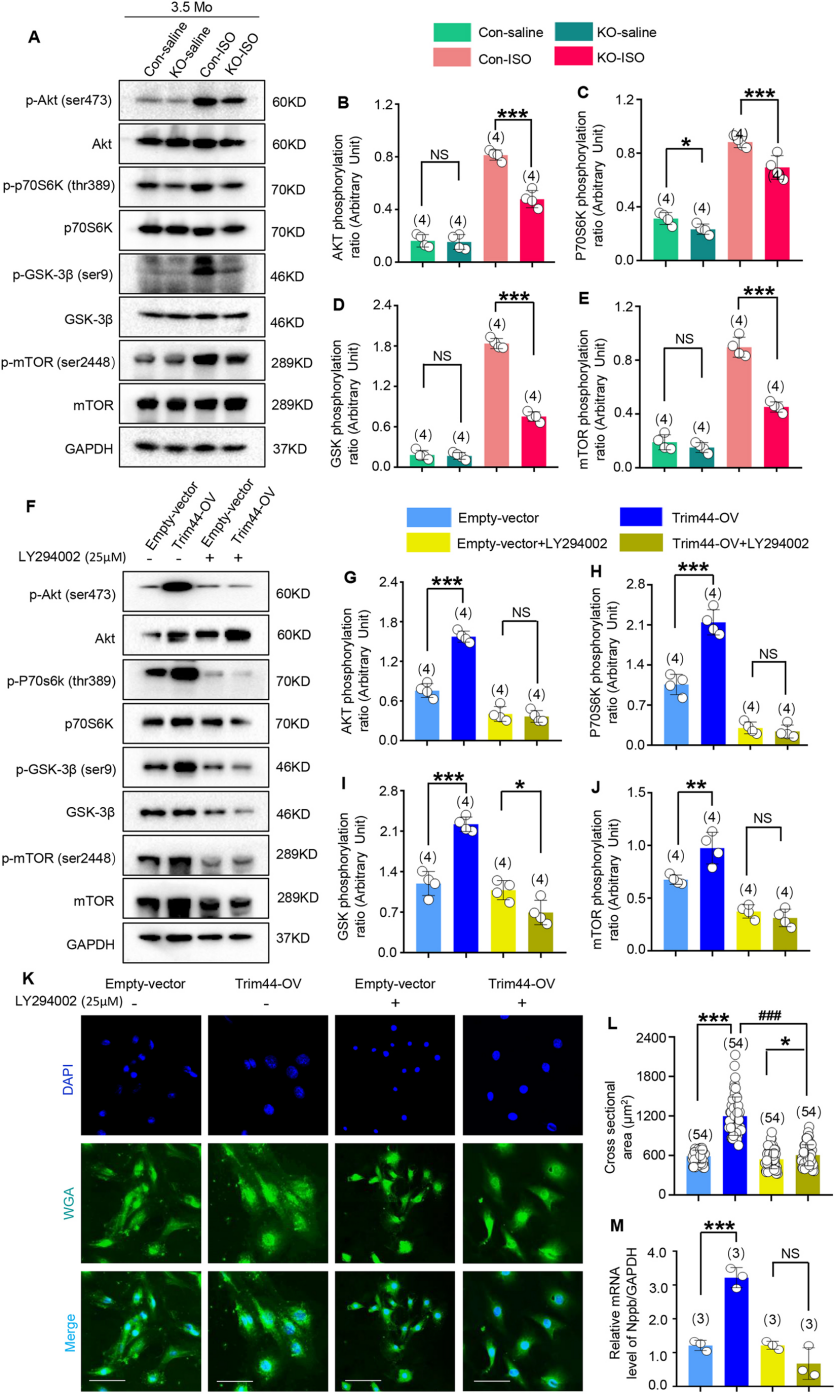
**Trim44 KO attenuates ISO-induced cardiac remodeling through inhibition of the AKT/mTOR pathway.** (A-E) Expression of phosphorylated (p-) and total AKT, P70S6K, GSK3β and mTOR in heart tissue from control-saline, KO-saline, control-ISO and KO-ISO groups 2 weeks after cessation of ISO treatment, detected using western blotting (A) and quantitatively analyzed using the respective total protein for normalization (B-E) (*n*=4 rats per group). (F-J) Expression of phosphorylated and total AKT, P70S6K, GSK3β and mTOR in H9c2 cells from the empty-vector group and Trim44 overexpression group (referred as Trim44-OV) with or without inhibitor treatment (PI3K/AKT inhibitor, LY294002; 25 μM), detected using western blotting (F) and quantitatively analyzed using the respective total protein for normalization (G-J) (*n*=4 replicates per group). (K) WGA immunofluorescence (scale bars: 40 μm). (L) Quantitative analysis of cross-sectional area of H9c2 cells for four groups (*n*=3-4 replicates per group, 54 cells per group). (M) Quantitative analysis of *Nppb* expression through real-time PCR for four groups, using *Gapdh* for normalization (*n*=3 replicates per group). *P<0.05, **P<0.01, ***P<0.001, ^###^*P*<0.001 versus respective control group (one-way ANOVA). NS, not significant.

Several investigations found that TRIM44 facilitates tumor development through activation of the AKT/mTOR signaling pathway, including phosphorylated P70S6K (also known as RPS6KB1) in several cases (Robins et al., 2014; Ong et al., 2014; Xing et al., 2016; Tan et al., 2017; Xiong et al., 2018; Zhou et al., 2019; Li et al., 2019; Wei et al., 2019; Ji et al., 2020; Qing et al., 2021). Multiple intracellular signaling pathways participate in cardiac hypertrophy, including PI3K/AKT, EGFR/MAPK and GP130/JAK/STAT3 (Frey and Olson, 2003; Lina et al., 2019; Hui et al., 2019). Therefore, we detected the activation of those signaling pathways in heart tissues from *Trim44* KO rats, especially under the pathological stimulus of cardiac hypertrophy/HF. We found that Trim44 deficiency had no effect on activities of the MAPK or STAT signaling pathways (Fig. S3). However, the inhibitory effects of Trim44 KO on pathological cardiac hypertrophy induced by ISO treatment were exerted by suppressing the AKT/mTOR/GSK3β/P70S6K signaling pathway (Fig. 6).

It is suggested that AKT activation has a direct influence on pathogenesis of cardiac hypertrophy (Abeyrathna and Su, 2015). In our study, the level of phosphorylated AKT protein increased obviously in heart tissues with ISO treatment (approximately sixfold). In addition, the level of phosphorylated P70S6K (approximately threefold), phosphorylated GSK3β (approximately ninefold) and phosphorylated mTOR (approximately fivefold), which are downstream of the AKT signaling pathway, increased markedly in heart tissues with ISO treatment (Fig. 6A-E; *n*=4 rats in control-saline group and control-ISO group), which was consistent with the findings from previous studies (Tian et al., 2021). However, the phosphorylation levels of the abovementioned proteins increased to a lesser extent in *Trim44* KO rats after pathological stimulus via ISO (Fig. 6A-E; *n*=4 rats per group, *P*<0.001, KO-ISO versus control-ISO). Interestingly, only the level of phosphorylated P70S6K protein showed a decrease in heart tissues of *Trim44* KO rats at baseline (Fig. 6A,C; *n*=4 rats per group, *P*<0.05, KO-saline versus control-saline).

To verify the anti-hypertrophic effect of Trim44 KO, whether mediated through the AKT signaling pathway or not, we established a Trim44 overexpression H9c2 cell line. Trim44 protein increased 18-fold in our established Trim44 overexpression H9c2 cell line (referred to as Trim44-OV) compared with that in the empty-vector group (see Fig. S4).

Our results showed that the AKT/mTOR/GSK3β/P70S6K signaling pathway was markedly activated in Trim44-OV cells (Fig. 6F-J; *n*=4 replicates per group, *P*<0.01 or *P*<0.001, Trim44-OV group versus empty-vector group). Subsequently, we treated H9c2 cells with a PI3K/AKT inhibitor (LY294002; 25 μM) and found that the levels of the abovementioned phosphorylated proteins decreased significantly in both the empty-vector group and Trim44-OV group, and there were no differences between these two groups after treatment with the inhibitor (Fig. 6F-J; *n*=4 replicates per group, no significance, Trim44-OV+inhibitor group versus empty-vector+inhibitor group).

In addition, we observed changes in the morphology and level of markers of cardiac stress in Trim44-OV cells. The cross-sectional area, which was observed by WGA immunofluorescence, increased by 103.4% in the Trim44-OV group compared with that in the empty-vector group. However, it increased by only 11.06% in the Trim44-OV group compared with that in the empty-vector group after treatment with the inhibitor (Fig. 6K,L; 54 cells per group, *P*<0.001 or *P*<0.05, versus vector group with or without inhibitor). Meanwhile, the level of *Nppb* mRNA increased significantly in Trim44-OV cells but showed no difference between the Trim44-OV group with inhibitor treatment and empty-vector group with inhibitor treatment (Fig. 6M; *n*=3 replicates per group, *P*<0.001, Trim44-OV group versus empty-vector group with inhibitor).

Therefore, the results of signal transduction *in vivo* and reverse validation analyses *in vitro* together demonstrated that Trim44 deficiency attenuated ISO-induced pathological cardiac remodeling through blocking the AKT/mTOR/GSK3β/P70S6K signaling pathway.

## DISCUSSION

When pathological hypertrophy progresses to HF, the prognosis is often very poor. Therefore, it is important to find intervention targets that can inhibit or even reverse pathological cardiac hypertrophy. Borlepawar et al.'s study suggested that the expression of TRIM44 increased in a variety of heart diseases including HF (Borlepawar et al., 2019), and our group previously found that the expression of TRIM44 was upregulated in clinical samples from HCM patients and in hypertrophic rat myocardium samples induced by ISO or ANG II. These findings suggest that TRIM44 may be involved in the pathological process of myocardial hypertrophy. However, knowledge of the impact of TRIM44 on physiology and pathological cardiovascular progression remains very limited.

In the present study, we first generated a myocardium-specific Trim44 KO rat using CRISPR-Cas9 technology. This KO allowed us to study the effects of Trim44 deficiency on the heart specifically and exclude interference from other organs and tissues. Geometric and functional observations of the heart based on echocardiographic and histopathological analyses revealed that Trim44 KO suppressed the geometric development of the heart. We subsequently investigated the response of Trim44 KO rats to pathological hypertrophy induced by ISO. Interestingly, rats with Trim44 deficiency exhibited ameliorated cardiac pathological changes at global, organizational and molecular levels in response to pathological stress. Furthermore, Trim44 deficiency in the myocardium resulted in inhibition of activation of pathways involved in myocardial hypertrophy in response to pathological stress. Finally, reverse validation of signal pathways in the H9c2 cell line further confirmed that the regulation was related to at least the AKT/mTOR pathway.

It has been suggested that dysregulation of TRIM44 protein is associated with several diseases. TRIM44 promotes cell proliferation and metastasis in various carcinogeneses, such as renal cell carcinoma, breast cancer and melanoma (Yamada et al., 2020; Kashimoto et al., 2012; Xiong et al., 2018; Ji et al., 2020; Zhou et al., 2017; Yamada et al., 2017; Kawabata et al., 2017; Tan et al., 2017; Zhou et al., 2019; Li et al., 2019), and also shows promise as a potential prognostic predictor and a therapeutic target for patients with multiple tumors (Ong et al., 2013; Kawaguchi et al., 2017; Li et al., 2021; Lyu et al., 2021; Peters et al., 2010; Ong et al., 2014; Chen et al., 2021; Liu et al., 2019; Robins et al., 2014; Sato et al., 2021). In addition, TRIM44 has also been reported to participate in neuron differentiation, neuron maturation (Kashevarova et al., 2014) and aniridia (Zhang et al., 2015a; Samant et al., 2016; Lim et al., 2017; Landsend et al., 2018). To the best of our knowledge, this is the first study to demonstrate the effects of Trim44 on the heart at baseline and under pathological stress.

The overall heart of the Trim44 KO rats became smaller, with morphological changes occurring from 3 months of age, and exhibited typical characteristics at 5 months of age, including thinning of the ventricular wall, reduction of the ventricular cavity, decreased LV mass and decreased SV. Subsequently, ISO was administered to induce pathological cardiac hypertrophy, and the deletion of Trim44 significantly inhibited the development of pathological hypertrophy and HF, including by improving overall heart morphology and echocardiographic parameters reflecting function, as well as by reducing myocardial fibrosis and hypertrophic cardiac cell size. In addition, the expression of Nppb, a molecular marker of cardiac dysfunction and HF, was also downregulated significantly. The above results all suggested that deletion of Trim44 may significantly affect the growth and development of the adult heart, especially through pathological cardiac hypertrophy.

Although the KO rats had phenotypic changes before ISO treatment, they were still selected as the control group for the study of ISO treatment. First, the effects of Trim44 KO on the morphology and function of the heart were progressive. Moreover, the *Trim44* KO rats exhibited typical characteristic phenotypes at 5 months of age, including thinning of the ventricular wall, reduction of the ventricular cavity, decreased LV mass and decreased SV. The cardiac phenotypes in *Trim44* KO rat at 3.5 months of age (end of the observation) were in an early stage of phenotypic development.

To better analyze the phenotypes induced by ISO treatment, we compared the echocardiographic parameters in KO-ISO and control-ISO groups before and after the ISO treatment (Fig. 4; Table S6), not just the absolute values at the end of the observation. Analysis in this way could rule out the effects of phenotypic changes in our KO animals prior to ISO treatment.

Multiple intracellular signaling pathways participate in cardiac hypertrophy, including the IGF1R/PI3K/AKT, EGFR/MAPK, GP130/Jak/STAT3 and calcineurin/NFAT pathways (Frey and Olson, 2003). Several investigators found that Trim44 facilitated tumor development through activation of the AKT/mTOR signaling pathway, including phosphorylated P70S6K in several cases (Ong et al., 2014; Xing et al., 2016; Li et al., 2019; Wei et al., 2019). Researchers have speculated that TRIM44 might be a valuable biomarker for identifying patients who will respond to treatment with drugs targeting the PI3K–AKT–mTOR signaling cascade (Wei et al., 2019; Xiong et al., 2018; Tan et al., 2017; Ong et al., 2014; Robins et al., 2014; Huo and Dou, 2021). Thus, in the present study, we examined changes in the signaling pathways mentioned above. As expected, the inhibitory effects of Trim44 KO on pathological cardiac hypertrophy induced by ISO treatment were exerted by suppressing the AKT/mTOR/GSK3β/P70S6K signaling pathway.

Studies have shown that the AKT signaling pathway plays a significant role in both physiological and pathological cardiac hypertrophy, and is a critical target in determining the severity of the pathological process (Shimizu and Minamino, 2016; Kemi et al., 2008; Dorn and Force, 2005). mTOR and GSK3β, downstream molecules of AKT, are phosphorylated by the activation of AKT (Matsui and Rosenzweig, 2005). Studies have shown that inhibition of mTOR with rapamycin treatment attenuates the hypertrophic response in samples from humans and in animal models (Shioi et al., 2003; McMullen et al., 2004; Soesanto et al., 2009). It has been suggested that GSK3β is an endogenous negative regulator of cardiac hypertrophy, the activity of which is strictly controlled by its phosphorylation, and inhibition of Ser9 phosphorylation in GSK3β reduces cardiac hypertrophy and risk of HF (Hardt and Sadoshima, 2004; Matsuda et al., 2008). In addition, further studies have suggested that P70S6K is also a crucial mediator of hypertrophy downstream of the AKT/mTOR cascade (Shioi et al., 2003; McMullen et al., 2004; Shiojima et al., 2005).

When we explored the molecular mechanism, we found no significant changes in key molecules in the MAPK and STAT signaling pathways (see Fig. S3). However, the expression of phosphorylated P70S6K decreased significantly in KO rats at baseline, indicating activation of the AKT/mTOR/GSK3β/P70S6K signaling pathway. Previous studies have shown that P70S6K plays an important role in the regulation of cardiac hypertrophy (Minamino et al., 2000; Boluyt et al., 1997; Sanada et al., 2002). Therefore, we hypothesized that the cardiac phenotypes due to Trim44 KO at baseline are at least partially induced by P70S6K.

AMP-activated protein kinase (AMPK; also known as PRKAA2) and AKT are two primary effectors in response to metabolic stress. AMPK is a stress-activated kinase that functions as a cellular fuel gauge and master metabolic regulator (Zungu et al., 2011). Physiological processes in which the PI3K/Akt/mTOR/P70S6K signaling pathway is involved include angiogenesis, hypoxic energy metabolism, insulin receptor signal transduction, protein metabolism and autophagy. The main signaling pathways in which P70S6K is involved include the AKT/mTOR signaling pathway and AMPK signaling pathway (Minamino et al., 2000; Matsui et al., 2005; Yang et al., 2020). One possible reason why the AKT/mTOR signaling pathway did not change might be that the KO efficiency of Trim44 protein in our model is not 100%; therefore, no statistical difference in the expression of the key molecules mentioned above was observed at baseline. In addition, upstream of P70S6K might be PI3K–PDK1, which is activated by growth factors, hormones, cytokines, etc. This provides an important clue for signal transduction analysis, which can be systematically analyzed and verified in subsequent research. However, when given a pathological stimulus, the inhibition of myocardial hypertrophy signals with Trim44 KO is clearly apparent, suggesting that Trim44 may mainly be involved in the regulation of pathological myocardial hypertrophy.

Because the deletion of Trim44 suppressed the AKT/mTOR signaling pathway, we subsequently constructed a cell line with Trim44 overexpression to verify the influence of high expression of Trim44 on the above signaling pathways *in vitro*. Our results showed that overexpression of Trim44 in the H9c2 cell line significantly activated the AKT/mTOR signaling pathway. The activation of the above pathways and the increased cross-sectional area of cardiomyocytes induced by Trim44 overexpression were significantly inhibited by the administration of PI3K/AKT inhibitor. These results indicate that the inhibitory effects of Trim44 KO on ISO-induced pathological myocardial hypertrophy were achieved, at least in part, through inhibition of the AKT/mTOR signaling pathway.

The expression of Trim44 is related to activity of the AKT/mTOR signaling pathway, and Trim44 knockdown inhibits activation of the AKT signaling pathway (Robins et al., 2014; Ong et al., 2014; Xing et al., 2016; Tan et al., 2017; Xiong et al., 2018; Zhou et al., 2019; Li et al., 2019; Wei et al., 2019; Ji et al., 2020; Qing et al., 2021). These findings are based on tumor research and related database analysis; however, the regulatory effects of Trim44 on the AKT/mTOR signaling pathway in myocardium have not yet been reported. Several reports have shown that Trim44, as a deubiquitinase, can stabilize the target protein through its deubiquitination, including Terf, hypoxia inducible factor-1α (HIF-1α) and Toll-like receptor 4 (TLR4) (Wei et al., 2019; Urano et al., 2009; Kashimoto et al., 2012; Yang et al., 2013; Chen et al., 2019). It has also been reported that TLR4 plays an important role in the regulation of cardiac hypertrophy, and that TLR4 participates in cardiac hypertrophy regulation through the mTOR signaling pathway (Yun et al., 2018; Tuan et al., 2005). Therefore, it is speculated that Trim44 may regulate the ubiquitination level of TLR4 in myocardium, affecting the activation state of the AKT/mTOR signaling pathway and thus participating in the regulation of cardiac hypertrophy.

If the reverse authentication was performed *in vivo* in an animal model, the conclusions would be more convincing, which is the shortcoming of this study. Therefore, subsequent research should be performed with more model types and under multiple pathological conditions, which could enable clearer understanding of the biological functions of Trim44, especially the regulation of cardiac development and cardiac pathological processes.

## MATERIALS AND METHODS

### Animals

All Sprague-Dawley (SD) rats used in this study were raised in facilities certified by the Association for the Assessment and Accreditation of Laboratory Animal Care (AAALAC). The breeding environment was maintained at a barrier environment standard, and light was alternated with dark for 12 h. The animals and related operating procedures involved in this study meet the requirements of animal welfare, follow the 3R principle and are approved by the Laboratory Animal Use and Management Committee (IACUC) of the Institute of Laboratory Animals Science, Chinese Academy of Medical Sciences and Peking Union Medical College, Beijing, China (ZLF18003).

The *α-MHC-Cre* transgenic rats were established in our laboratory as previously reported (Ma et al., 2017). Cre-specific primers (*Cre* F, 5′-AACATGCTTCATCGTCGGTC-3′; *Cre* R, 5′-GTGCCTTCTCTACACCTGCG-3′) were used for genotyping through PCR, and the identified transgenic positive rats were maintained by mating with SD WT rats. *Trim44* cKO was established in our laboratory. A pair of synthetic oligonucleotides for sgRNA (sgRNA_1_, CCTTGCCGCTTTAAGTGACTC; sgRNA_2_, CCATGTTGGGAGCATTGCCTA) were annealed and then cloned into the pUC57-sgRNA expression vector, and the floxed plasmid donor was cloned into the pGSI plasmid. Both the Cas9 and sgRNA expression plasmids were linearized and used as templates for *in vitro* transcription. A mixture of the donor vector (4 ng/µl), Cas9 mRNA (25 ng/µl), and sgRNAs (10 ng/µl each) was microinjected into both the cytoplasm and male pronucleus of the fertilized eggs (Ma et al., 2014). The injected zygotes were then transferred to pseudopregnant SD rats, which then carried them to parturition. The genome was prepared according to standard procedures as previously reported (Ma et al., 2014; Wu et al., 2020), and specific PCR primer sequences for *Trim44* cKO were as follows: *Trim44* flox F, 5′-ACTTTTCCTCTGCCCCACTAGATC-3′; *Trim44* flox R, 5′-CCACTTTACCCACGCCGTC-3′. The *Trim44* cKO was maintained by mating with SD WT rats. First, we crossed the α*-MHC-Cre* tool rat with the *Trim44* cKO rat, then we acquired rats with the genotype of both the *Trim44*^flox/+^ and *α-MHC-Cre* transgenic positive (referred as *Trim44*^flox/+^/*α-MHC-Cre*). In the next round of breeding, we crossed the *Trim44*^flox/+^/*α-MHC-Cre* rat with the *Trim44* cKO rat, then the pups with the genotype of both the *Trim44*^flox/flox^ and *α-MHC-Cre* transgenic positive (referred as *Trim44*^flox/flox^/*α-MHC-Cre*) were used in subsequent studies and referred as *Trim44* KO rats.

### Human cardiac samples

Cardiac samples from patients with HCM who underwent morrow septal myectomy were used for expression analysis. Here, we purposely chose patients without MI, valvular heart disease or any other congenital heart disease, in order to minimize the interference effects. Normal control samples were obtained from the same cardiac region in healthy donors. Informed consent was obtained from participants or their relatives. Sample use and the research plan were approved by the Ethics Committee of Fuwai Hospital. All clinical investigation was conducted according to the principles expressed in the Declaration of Helsinki.

### ISO treatment

SD rats in the KO-ISO and WT-ISO groups were administered ISO (4.5 mg/kg body weight; in saline) subcutaneously at 2.5 months of age, and rats in the KO-saline and WT-saline groups were treated with an equal volume of saline at the same age. The ISO was freshly prepared and given once daily for 14 days. Moreover, 2 weeks after cessation of ISO treatment, rats were subjected to echocardiography and then sacrificed. The hearts were excised and washed carefully in ice-cold saline, blotted dry with tissue paper and weighed. Heart tissues were then fixed with formalin or snap frozen in liquid nitrogen for subsequent analysis.

### ANG II treatment

SD WT rats were anesthetized by isoflurane (1.5-2.5%), then an incision was made in the midscapular region, and the osmotic minipumps (model 2004, ALZET, Cupertino, CA, USA) containing ANG II (0.3 mg/kg/day; Merck, Kenilworth, NJ, USA) were implanted. Rats in the sham group underwent the same surgical procedure with an empty osmotic pump. Echocardiography was performed 28 days after pump implantation, and the hearts were excised, washed carefully in ice-cold saline, and then blotted dry with tissue paper or snap frozen in liquid nitrogen for subsequent analysis.

### Echocardiography

Echocardiography was performed using a Vevo3100 system (FUJIFILM, VisualSonics, Toronto, Canada). Rats were anesthetized by isoflurane (1.5-2.5%), and their body temperature and heart rate were maintained during echocardiographic inspection. The measurements were recorded as the average of at least three consecutive cardiac cycles. The LVDS, LVDD, LVPWS, LV posterior wall thickness at end diastole (LVPWD), SV, LVFS and LV mass were analyzed with Vevo LAB 5.5.0 (Ling et al., 2021).

### Histological analysis

For light microscopy, rat heart tissue was fixed in formalin overnight at room temperature and then embedded in paraffin. The paraffin sections were cut at 4-6 μm thickness and subjected to H&E staining or Masson's trichrome staining, which was performed as previously reported (Wu et al., 2020; Lu et al., 2018). The stained sections were analyzed with Aperio ImageScope v8.2.5 software (Guan et al., 2019; Lu et al., 2012).

### Immunohistochemistry

Paraffin sections of heart tissues were prepared according to standard pathological procedures as previously described (Lu et al., 2014). The paraffin-embedded sections were first dewaxed and rehydrated, and then reagents were applied for tissue antigen retrieval. The sections were blocked (ZLI-9056, ZSGB-BIO, Beijing, China) and incubated with anti-Trim44 antibody (11511-1-AP, Proteintech, Rosemont, IL, USA; 1:50) overnight at 4°C. Then, the sections were washed with PBS and incubated with an anti-rabbit horseradish peroxidase-conjugated secondary antibody for 1 h at room temperature. After incubation with DAB solution (ILI-9017, ZSGB-BIO), the slides were counterstained with Hematoxylin. The slice images were collected and analyzed by a scanning system (Pannoramic 250 FLASH, 3DHISTECH, Budapest, Hungary).

### Cell culture and establishment of Trim44 overexpression cell line

The rat embryonic ventricular myocyte cell line H9c2 was grown in high-glucose DMEM (C11995500BT, Gibco, Beijing, China), supplemented with 10% fetal bovine serum (11011-B611, TIANHANG, Hangzhou, China), 100 U/ml penicillin and 100 g/ml streptomycin, in an incubator at 37°C (5% CO_2_ and 95% air). Trypsin (T1320, Solarbio, Beijing, China) digestion was carried out for cell passage. The expression construct for Trim44 was generated by cloning full-length human *TRIM44* cDNA fragment into the pENTER vector (WZ Biosciences, Jinan, China). H9c2 cells were transfected with the Trim44 plasmid using Lipofectamine 2000 transfection reagent (11668019, Thermo Fisher Scientific, Waltham, MA, USA), and the establishment of stable cell lines was completed by selection with 1 μg/ml puromycin (Thermo Fisher Scientific). The cells were treated with LY294002 (9901, Cell Signaling Technology, Danvers, MA, USA; 25 μM) for 12 h. Finally, cells were prepared for immunofluorescence, western blotting or RT-PCR analysis.

### Immunofluorescence

The paraffin sections of heart tissues were prepared according to standard procedures as previously described (Wu et al., 2020; Lu et al., 2018, 2014). The sections were first dewaxed and rehydrated, and then reagents were applied for tissue antigen retrieval. The sections were blocked and incubated with anti-Trim44 antibody (11511-1-AP, Proteintech; 1:50), anti-cardiac troponin T (cTnT) antibody (MA5-129060, Thermo Fisher Scientific; 1:40) or anti-WGA antibody (W7024, Thermo Fisher Scientific; 1:200) overnight at 4°C. Then, they were washed with PBS and incubated with Alexa Fluor 488-conjugated goat anti-mouse IgG (A11029, Thermo Fisher Scientific; 1:200) or Alexa Fluor Plus 555-conjugated goat anti-rabbit IgG (A32732, Thermo Fisher Scientific; 1:200) for 1 h at room temperature. For cell slides, H9c2 cells were cultured as described above and treated under standard procedures as previously described (Lu et al., 2016). Then, the slides were incubated with the anti-WGA antibody (W7024, Thermo Fisher Scientific; 1:200) overnight at 4°C. All slides were counterstained with 4′6-diamidino-2-phenylindole (DAPI; ZLI-9557, ZSGB-BIO). Finally, the slice images were collected and analyzed by a scanning system (Pannoramic 250 FLASH, 3DHISTECH).

### Protein extraction and immunoblotting

Protein lysates of rat heart tissue were prepared according to the instructions of the supplier (78510, 78420, 87785, Thermo Fisher Scientific). The proteins were separated by SDS-PAGE and then transferred to nitrocellulose membranes. The membranes were incubated overnight in the hybridization bag with antibodies against Trim44 (11511-1-AP, Proteintech; 1:500), phosphorylated (4060, Cell Signaling Technology; 1:2000) or total (4691, Cell Signaling Technology; 1:2000) AKT, phosphorylated (5536, Cell Signaling Technology; 1:1000) or total (2983, Cell Signaling Technology; 1:1000) mTOR, phosphorylated (9204, Cell Signaling Technology; 1:2000) or total (2708, Cell Signaling Technology; 1:2000) p70S6K, or phosphorylated (5558, Cell Signaling Technology; 1:2000) or total (9832, Cell Signaling Technology; 1:2000) GSK3β. Then, antibody binding was detected with the corresponding secondary antibody (ZSGB-BIO). Finally, the membranes were exposed to enhanced chemiluminescence reagent and visualized with an XRS+ Gel Imaging System (Bio-Rad, Hercules, CA, USA). GAPDH or β-actin (Proteintech; 1:10,000) was used for normalization. Quantitative analysis was completed with ImageJ software.

### RNA extraction, quantification and RT-PCR

Total RNA was isolated from rat heart tissue or H9c2 cells using TRIzol Reagent (15596018, Thermo Fisher Scientific) as previously reported (Lu et al., 2014, 2018). First-strand cDNA was obtained according to the manufacturer's protocol (AU341-02, Transgen, Beijing, China). The PCR reactions were amplified and analyzed using a QuantStudio 3 Real-Time PCR System (Thermo Fisher Scientific). The levels of *Nppb* mRNA were detected and normalized by *Gapdh* under standard conditions (*Gapdh* F, 5′-CTCATGACCACAGTCCATGC-3′; *Gapdh* R, 5′-TTCAGCTCTGGGATGACCTT-3′; *Nppb* F, 5′-TAGCCAGTCTCCAGAACAA-3′; *Nppb* R, 5′-AACAACCTCAGCCCGTCA-3′).

### Statistical analysis

Data in this study were analyzed with unpaired two-tailed Student's *t*-tests for two groups. Data are expressed as means±s.d. One-way analysis of variance (ANOVA; with Tukey correction) was performed for multiple groups. Differences were considered significant at *P*<0.05. Statistical analysis was performed using Prism software (GraphPad 8).

## Supplementary Material

Supplementary information
