# Supplementary material for: Cardiac-specific Trim44 knockout in rat attenuates isoproterenol-induced cardiac remodeling via inhibition of AKT/mTOR pathway
Source: Dis Model Mech. 2022 Aug 22;16(5):dmm049444. doi: 10.1242/dmm.049444 (PMC9441189; doi:10.1242/dmm.049444)
Supplement: Supplementary information [file dmm-16-049444-s1.pdf]

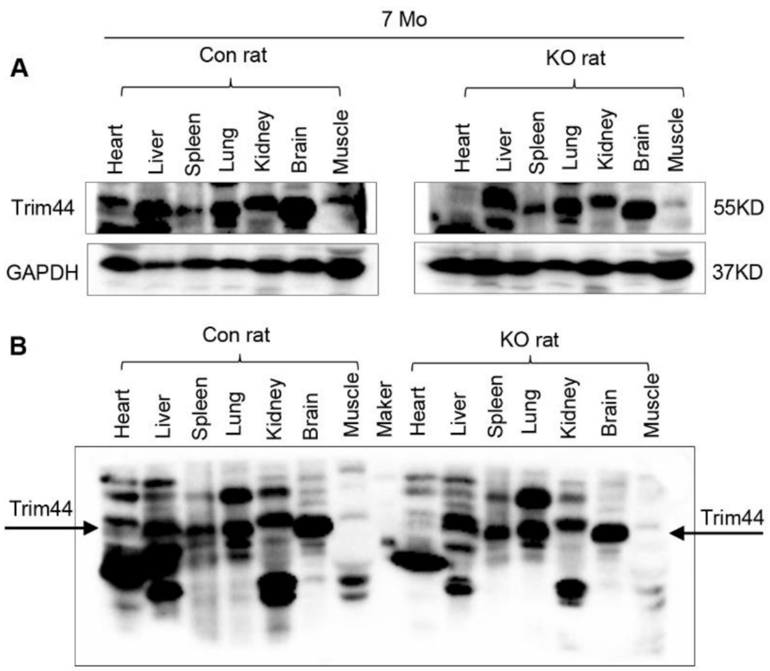

Fig. S1. (A) Expression of Trim44 in protein lysate from heart tissue, liver tissue, spleen tissue, lung tissue, kidney tissue, brain tissue and muscle tissue in control and KO rats, which was detected using western blot and using GAPDH for normalization. (n=3 replicates per group). (B) The immunoblotting was performed in single nitrocellulose membranes, in order to facilitate horizontal comparison between these two groups.

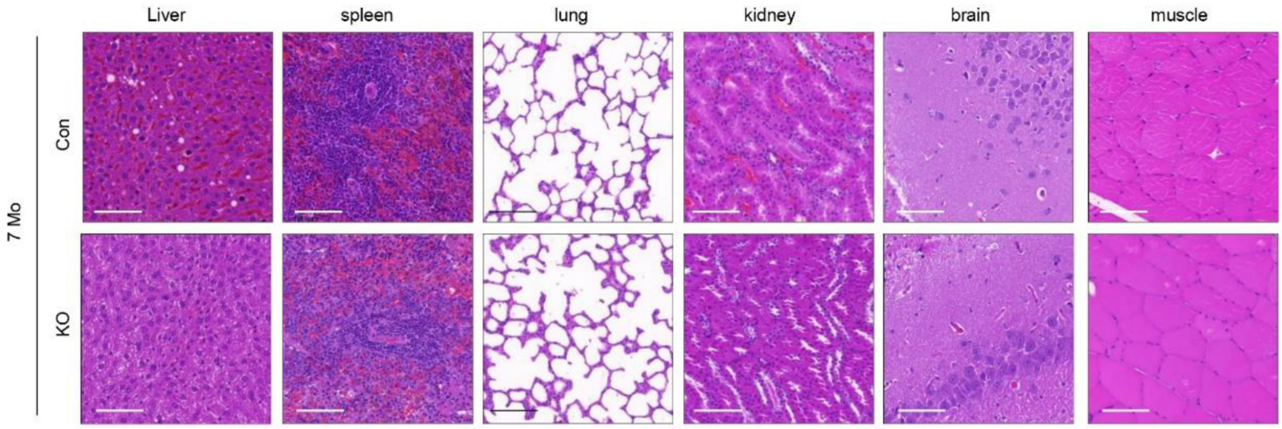

Fig. S2. Representative magnification screenshot with Hematoxylin and eosin (H&E) staining in liver tissue, spleen tissue, lung tissue, kidney tissue, brain tissue and muscle tissue from control and KO rats at 7 months of age (white scale bar=100µm).

Disease Models & Mechanisms • Supplementary information

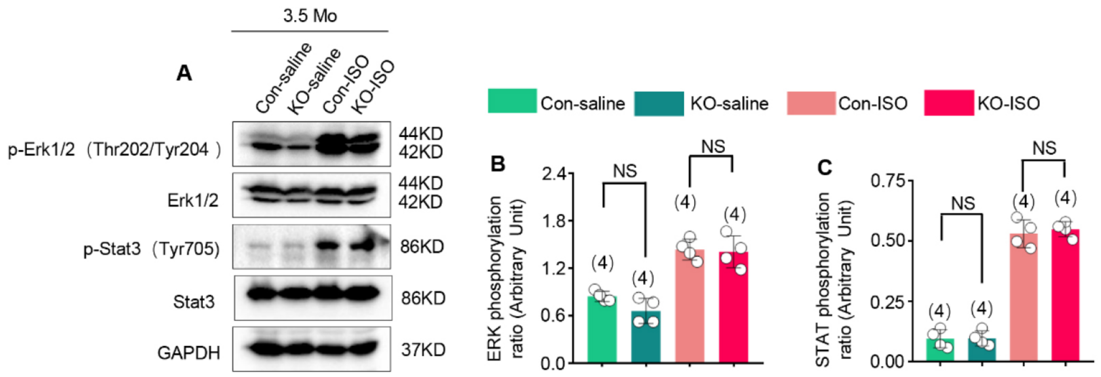

Fig. S3. (A) Expression of phosphorylated and total Erk1/2 and Stat3 in heart tissue from control-saline, KO-saline, control-ISO and KO-ISO group 2 weeks after cessation of ISO treatment, which was detected using western blot and was quantitatively analyzed using respective total protein for normalization (B-C, n=4 rats per group). There was no statistical difference between these two groups (NS).

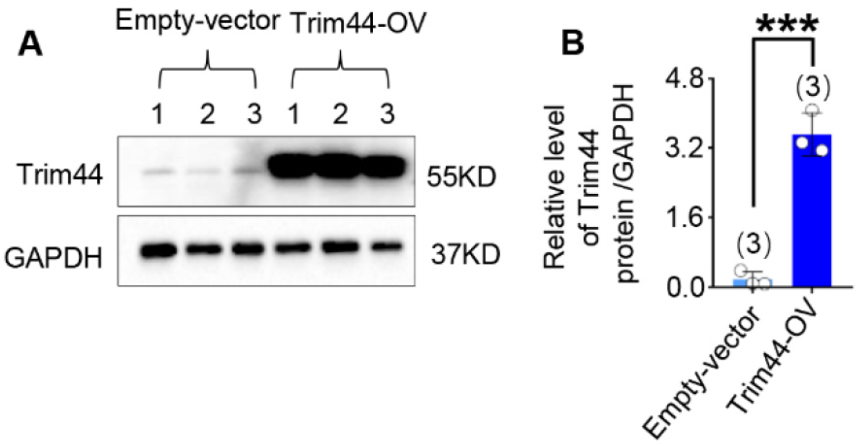

Fig. S4. (A) Overexpression efficiency of Trim44 in H9c2 cell line, which was detected using western blot and quantitatively analyzed using GAPDH for normalization (B, n=3 replicates per group). ****P*<0.001 *versus* empty-vector group.

Disease Models & Mechanisms • Supplementary information

Table S1. Echocardiographic parameters of *Trim44* KO rats at 1 month, 3 months, 5 months, 10 months and 15 months of age

Parameters	Units	1 month		3 months		5 months		10 months		15 months	
		Con	KO	Con	KO	Con	KO	Con	KO	Con	KO
		n=6	n=6	n=7	n=7	n=8	n=8	n=8	n=9	n=9	n=11
LVDS	mm	5.16±0.82	4.35±0.47	5.26±0.70	4.37±0.85	5.36±0.66	4.54±0.77*	5.43±0.68	4.65±0.69*	5.60±0.59	5.00±0.54*
LVDD	mm	8.23±0.63	7.49±0.58	8.46±0.60	7.57±0.87	8.55±0.61	7.71±0.67*	8.58±0.65	7.75±0.78*	8.64±0.51	7.87±0.78*
LVPWS	mm	1.14±0.14	0.94±0.19	1.18±0.10	1.03±0.13*	1.46±0.31	1.08±0.13**	2.30±0.38	2.06±0.36	2.20±0.47	1.89±0.34
LVPWD	mm	0.95±0.29	0.75±0.14	1.04±0.20	0.81±0.13*	1.08±0.10	0.87±0.11**	1.22±0.19	1.08±0.19	1.17±0.24	1.01±0.17
SV	mm^3	250.42±35.07	212.67±22.82	251.62±35.25	216.74±41.37	272.77±36.01	220.68±36.10*	284.88±53.05	230.10±40.49*	303.00±73.10	238.10±34.92*
LV Mass	g	454.67±71.81	313.06±66.30*	489.18±80.31	322.20±53.55**	524.09±97.79	327.91±93.65**	587.54±98.45	403.14±94.50**	593.04±86.90	423.20±84.57**
FS	%	28.53±1.59	30.91±4.74	31.48 ±6.61	31.79 ±4.98	32.65±5.87	28.25±3.23	42.28±3.55	39.52±6.55	37.62±6.48	35.65±6.40

Con, Control; LVDS, left ventricular (LV) diameter at end systolic; LVDD, LV diameter at end diastolic; LVPWS, LV posterior wall thickness at end systolic; LVPWD, LV posterior wall thickness at end diastolic; SV, stroke volume; FS, fractional shortening.
*$P<0.05$, **$P<0.01$ *versus* control group.

Table S2. HW/BW of *Trim44* knockout rats at 5 months of age

Parameters	Unit	Control (n=8)	KO (n=8)
BW	g	336.13±70.24	332.50±63.50
HW/BW	mg/mm	0.38±0.02	0.33±0.04**

BW, body weight; HW/BW, heart weight to body weight ratio. ***P*<0.01 *versus* control group.

Table S3. Echocardiographic parameters of *Trim44* knockout rats at 2 weeks of age

Parameters	Unit	Con (n=6)	KO (n=6)
BW	g	29.21±4.27	28.44±4.48
HW/TL	mg/mm	1.46±0.13	1.44±0.21
LVDS	mm	2.20±0.26	2.21±0.32
LVDD	mm	4.28±0.41	4.24±0.30
LVPWS	mm	0.93±0.11	0.90±0.17
LVPWD	mm	0.75±0.09	0.72±0.08
SV	mm^3	66.48±15.19	64.16±9.31
FS	%	48.43±4.29	48.02±5.30
LV Mass	g	95.96±18.38	86.75±14.75

BW, body weight; HW/TL, heart weight to tibial length ratio; LVDS, left ventricular (LV) diameter at end systolic; LVDD, LV diameter at end diastolic; LVPWS, LV posterior wall thickness at end systolic; LVPWD, LV posterior wall thickness at end diastolic; SV, stroke volume; FS, fractional shortening; LV Mass, left ventricular mass. There was no statistical difference in all parameters.

Table S4. Echocardiographic parameters of *Trim44* knockout rats at 7 months of age

Parameters	Unit	Con (n=4)	Trim44$^{flox/+}$/*α-MHC-Cre* (n=4)	Trim44$^{flox/flox}$/*α-MHC-Cre (reffered as KO)* (n=4)
BW	g	386.50±119.53	400.88±103.05	387.93±117.70
HW/TL	mg/mm	35.02±10.72	34.13±7.48	26.74±4.86
LVDS	mm	5.39±0.15	5.42±0.19	4.61±0.50*
LVDD	mm	8.56±0.68	8.58±0.38	7.72±0.08*
LVPWS	mm	2.13±0.42	2.24±0.56	2.01±0.19
LVPWD	mm	1.11±0.19	1.23±0.32	0.10±0.07
SV	mm^3	281.87±29.62	271.40±39.68	223.27±31.73*
FS	%	43.00±4.47	39.44±6.09	35.71±3.21*
LV Mass	g	579.48±85.39	594.81±128.10	380.93±88.26*
E/A		1.65±0.18	1.62±0.12	1.63±0.05

BW, body weight; HW/TL, heart weight to tibial length ratio; LVDS, left ventricular (LV) diameter at end systolic; LVDD, LV diameter at end diastolic; LVPWS, LV posterior wall thickness at end systolic; LVPWD, LV posterior wall thickness at end diastolic; SV, stroke volume; FS, fractional shortening; LV Mass, left ventricular mass; E/A, early diastolic filling to atrial filling velocity ratio of mitral flow. *P<0.05 *versus* control group.

Table S5. Coefficient of major organs of *Trim44* knockout rats at 7 months of age

Parameters	Unit	Con (n=4)	Trim44$^{flox/+}$/*α-MHC-Cre* (n=4)	Trim44$^{flox/flox}$/*α-MHC-Cre (reffered as KO)* (n=4)
Body Weight	g	386.50±119.53	400.88±103.05	392.93±113.58
Heart Weight/TL	mg/mm	37.04±8.80	34.13±7.48	23.66±1.81*
Liver Weight/TL	mg/mm	255.05±49.09	268.31±53.82	260.96±62.42
Slpeen Weight/TL	mg/mm	13.30±2.19	14.53±0.92	12.81±1.23
Lung Weight/TL	mg/mm	61.59±15.53	65.87±13.27	66.59±12.72
Kidney Weight/TL	mg/mm	61.88±13.96	60.31±11.05	60.46±9.97
Brain Weight/TL	mg/mm	44.16±3.06	42.03±4.36	45.33±2.04

TL, tibial length. *P<0.05 *versus* control group.

Table S6. Echocardiographic parameters of *Trim44* knockout rats at 2 weeks after cessation of ISO treatment

Parameters	Unit	Con-saline n=6	KO-saline n=6	Con-ISO n=6	KO-ISO n=5
LVDS	mm	5.29±0.44	4.40±0.62*	1.60±0.41###	2.79±0.82*
LVDD	mm	8.50±0.54	6.87±0.22	7.61±0.83	7.20±0.63
LVPWS	mm	1.20±0.08	1.04±0.12*	4.13±0.57###	3.13±0.20**
LVFS	%	32.86±6.12	31.17±4.17	76.82±5.64###	61.89±8.37***
SV	mm3	259.00±30.66	237.45±15.97	217.95±38.38	241.45±33.54

LVDS, left ventricular (LV) diameter at end systolic; LVDD, LV diameter at end diastolic; LVPWS, LV posterior wall thickness at end systolic; LVFS, LV fractional shortening; SV, stroke volume. *P<0.05, **P<0.01, ***P<0.001 *versus* Con-saline or Con-ISO; ###P<0.001 *versus* Con-saline.

Disease Models & Mechanisms • Supplementary information
